# Pseudo-teams in Healthcare: The Perils of Impersonating a Cohesive Unit

**DOI:** 10.12669/pjms.39.4.7665

**Published:** 2023

**Authors:** Shankar Lal, Ehtesham Khan

**Affiliations:** 1 Dr. Shankar Lal, FCPS, FCARCSI. Fellow Neuro-Anaesthesiology and Neuro-Critical Care Medicine, Beaumont Hospital, Dublin, Ireland; 2 Dr. Ehtesham Khan, MSc, FCPS, FCARCSI, FJFICMI, EDIC, DMMD, Dip Pain RCSI. Consultant, Department of Anaesthesiology and Critical Care Medicine, Our Lady of Lourdes Hospital, Drogheda, Ireland. Chair Credential committee and Council Member CAI

**Keywords:** Pseudo-teams, Team dynamics, Workplace culture, Patient Safety

## Abstract

Teamwork is a critical aspect of healthcare and is widely recognised as a cornerstone of effective patient care. However, not all healthcare teams are created equal, and some teams that appear to work well together on the surface are better described as pseudo-teams rather than true teams. This issue is frequently disregarded and overlooked despite its importance, yet it significantly impacts patient care and staff morale. We wish to describe the concept of pseudo teams in healthcare, their perils, and ways to create true teams characterised by shared goals, open communication, and a commitment to each other’s success.

## BACKGROUND

In healthcare, a “true team” refers to a cohort of professionals who collaborate cooperatively, intending to deliver exceptional patient care. This notion has received increasing recognition recently due to the growing awareness of the significance of interdisciplinary teamwork in healthcare.^1^

Healthcare organisations often utilise teams to deliver high-quality patient care and accomplish shared objectives. Teams in the healthcare sector come in various forms, including interdisciplinary teams, cross-functional teams, and project teams. Each has a distinct purpose and comprises individuals with diverse skill sets and responsibilities.

Interdisciplinary teams bring together healthcare professionals from different fields to collaborate in providing comprehensive care to patients. Such teams could include a patient care team comprising a physician, nurse, and social worker or a discharge planning team comprising a physician, nurse, discharge planner, and therapist.^2^

On the other hand, cross-functional teams consist of professionals from different departments within a healthcare organisation working together on a specific project or challenge, such as enhancing patient satisfaction, reducing readmissions, or streamlining processes.^3^ Project teams, assembled for a defined period, are created for a specific project or task and often involve members from multiple departments and external stakeholders.^4^

### What are Pseudo-teams in healthcare?

A pseudo-team is a group of individuals who work interdependently but lack the characteristics that define a true team. These characteristics include a shared sense of purpose, open communication, and a commitment to each other’s success. In healthcare, pseudo-teams can result in fragmented care, negatively impact the work environment, and lead to burnout and turnover among staff.^5^

### The Perils of Pseudo-teams in healthcare

***Fragmented Care:*** One of the main problems with pseudo-teams in healthcare is that they can lead to fragmented care. This occurs when team members work in silos and do not effectively coordinate their efforts. This can result in patients receiving inconsistent or conflicting information and treatments, leading to confusion and potentially harmful outcomes.^6^

### Negative Work Environment:

In a true team, members support each other and work together to overcome challenges. In a pseudo-team, however, team members may compete with each other or engage in blame-shifting, leading to a hostile and stressful work environment. This can result in high burnout and turnover among healthcare staff, negatively impacting patient care.^7^

### Creating True Teams in Healthcare

True teamwork is a crucial driver of high-quality, patient-centred care in the healthcare organisations. Effective teamwork can improve patient outcomes, increase efficiency, and enhance job satisfaction among healthcare workers. However, creating true teams that can work together seamlessly and achieve shared goals can be challenging.^8^,^9^

Given the challenges of creating true teams in healthcare, it is crucial for healthcare organisations to adopt strategies to promote teamwork and collaboration among their staff. Some evidence-based strategies include:

1. ***Fostering a Team-Oriented Culture:*** Promoting a shared understanding of team goals, open communication, and recognition and reward of teamwork can create a culture of teamwork.^10^
2. ***Encouraging Interprofessional Collaboration:*** Cross-disciplinary training, opportunities for cross-disciplinary collaboration, and team-based problem-solving can help break down barriers to teamwork and enhance interprofessional collaboration.^11^
3. ***Improving Communication:*** Encouraging open and transparent communication, fostering a culture of trust, and providing training and resources to support practical communication skills can improve communication among team members.
4. ***Providing Ongoing Training and Support:*** Regular team meetings, training on teamwork and collaboration skills, and resources and tools to support teamwork can ensure that teams remain effective over time.^12^
5. ***Defined Roles and Responsibilities:*** A clear understanding of individual roles and responsibilities within the team is crucial in reducing confusion and promoting accountability. This can be achieved through formal job descriptions and regular performance evaluations.^13^
6. ***Mutual Respect and Trust:*** Teams that exhibit trust and mutual respect are more likely to work together effectively. Team members should feel comfortable expressing their opinions and providing feedback to their colleagues. A supportive and inclusive team culture, where all team members feel valued, is critical in promoting mutual respect and trust.^13^
7. ***Recognition and Rewards:*** Recognising and rewarding team members for their contributions can improve morale and encourage teamwork. This can take the form of formal recognition programs, such as employee-of-the-month awards, or informal expressions of gratitude, such as thank-you notes or team lunches.^14^
8. ***Leadership Support:*** Effective leadership is critical for developing true teams in healthcare settings. Leaders should provide clear direction, support team members, and create a positive team culture. Regular communication with team members, providing opportunities for professional development, and promoting a supportive and inclusive team environment are critical components of effective leadership.^14^

### The advantages of a true-team to an organisation:

- Streamlined efficiency and output through seamless coordination and cooperation.
- Improved decision-making
- A boost in job satisfaction and employee morale results in a reduction in turnover and absenteeism.
- Heightened adaptability and creativity facilitated by frequent communication and harmonious goals.
- Heightened patient satisfaction is attributed to consistent, excellent service delivery.

### The perks of a true-team to team itself:

- A clear definition of roles and responsibilities leads to heightened accountability and performance.
- Strengthened trust and communication among team members.
- Increased motivation and engagement due to shared achievements and professional growth opportunities.
- Elevated problem-solving and creativity through varied perspectives and open communication.
- A boost in team unity and a sense of belonging, fostering a positive work environment.

### The benefits of a dedicated team to a team member:

- An elevated level of job satisfaction through a positive work environment and shared achievements.
- Professional advancement through opportunities for skill acquisition and mentorship.
- A heightened sense of purpose and motivation due to shared goals and a well-defined understanding of roles and responsibilities.
- An improvement in work-life balance through a well-coordinated and delegated task structure.
- Strengthened job security through a stable and efficient team organisation.

### The advantages of a true team to patients:

- A heightened quality of care through improved coordination and communication among healthcare providers.
- Faster and more accurate diagnoses and treatment plans resulting from the collective expertise and problem-solving abilities.
- Improved patient satisfaction through consistent, high-quality care.
- Elevated safety through regular evaluations and improvements in processes and procedures.
- Enhanced continuity of care through accurate documentation and communication among healthcare providers.

## CONCLUSION

Pseudo-teams in healthcare can have serious consequences for staff and patients. Increasing awareness about pseudo-team culture in healthcare and emphasising its negative consequences is essential. Organisations must strive to establish clear goals and expectations for the team to avoid pseudo-teams’ dangers and create true teams in healthcare. This includes ensuring that team members understand their purpose and how they will work together to achieve it. Additionally, healthcare organisations can foster open communication and collaboration by providing opportunities for team members to discuss their work and collaborate on projects. The benefits of a true team are that improved collaboration and problem-solving lead to increased job satisfaction and better decision-making. When individuals in a true team establish trust and support for one another, they can work together seamlessly, generating exceptional outcomes for the team and the organisation. True teams also cultivate a sense of community and shared purpose, elevating the work environment and boosting employee engagement and retention.

### Authors Contribution:

**SL and EK:** Literature review and write up.
